# Precise individual muscle segmentation in whole thigh CT scans for sarcopenia assessment using U-net transformer

**DOI:** 10.1038/s41598-024-53707-8

**Published:** 2024-02-08

**Authors:** Hyeon Su Kim, Hyunbin Kim, Shinjune Kim, Yonghan Cha, Jung-Taek Kim, Jin-Woo Kim, Yong-Chan Ha, Jun-Il Yoo

**Affiliations:** 1 Department of Biomedical Research Institute, Inha University Hospital, Incheon, South Korea; 2 Department of Orthopaedic Surgery, Daejeon Eulji Medical Center, Daejeon, South Korea; 3 Department of Orthopedic Surgery, Ajou University School of Medicine, Suwon, South Korea; 4 Department of Orthopaedic Surgery, Nowon Eulji Medical Center, Seoul, South Korea; 5 Department of Orthopaedic Surgery, Seoul Bumin Hospital, Seoul, South Korea; 6 Department of Orthopedic Surgery, School of Medicine, Inha University Hospital, Incheon, South Korea

**Keywords:** Sarcopenia, Computed tomographic, Thigh, Muscles, Hip fracture, Medical research, Engineering, Mathematics and computing, Anatomy, Musculoskeletal system

## Abstract

The study aims to develop a deep learning based automatic segmentation approach using the UNETR(U-net Transformer) architecture to quantify the volume of individual thigh muscles(27 muscles in 5 groups) for Sarcopenia assessment. By automating the segmentation process, this approach improves the efficiency and accuracy of muscle volume calculation, facilitating a comprehensive understanding of muscle composition and its relationship to Sarcopenia. The study utilized a dataset of 72 whole thigh CT scans from hip fracture patients, annotated by two radiologists. The UNETR model was trained to perform precise voxel-level segmentation and various metrics such as dice score, average symmetric surface distance, volume correlation, relative absolute volume difference and Hausdorff distance were employed to evaluate the model’s performance. Additionally, the correlation between Sarcopenia and individual thigh muscle volumes was examined. The proposed model demonstrated superior segmentation performance compared to the baseline model, achieving higher dice scores (DC = 0.84) and lower average symmetric surface distances (ASSD = 1.4191 ± 0.91). The volume correlation between Sarcopenia and individual thigh muscles in the male group. Furthermore, the correlation analysis of grouped thigh muscles also showed negative associations with Sarcopenia in the male participants. This thesis presents a deep learning based automatic segmentation approach for quantifying individual thigh muscle volume in sarcopenia assessment. The results highlights the associations between Sarcopenia and specific individual muscles as well as grouped thigh muscle regions, particularly in males. The proposed method improves the efficiency and accuracy of muscle volume calculation, contributing to a comprehensive evaluation of Sarcopenia. This research enhances our understanding of muscle composition and performance, providing valuable insights for effective interventions in Sarcopenia management.

## Introduction

Sarcopenia, a condition characterized by age-related loss of muscle mass, strength and function, has garnered significant attention in recent years. As research progresses, there has been a shift in the consensus and focus from solely considering total muscle volume to exploring muscle quality and performance factors^1^. Understanding the intricate relationship between muscle composition, function and overall health has become crucial in developing effective interventions for individuals at risk of or affected by sarcopenia^2^.

The diagnostic consensus of Sarcopenia has been revised in recent years to improve accuracy and standardization. In 2019, known as EWGSOP2 and AWGS2019, introduced diagnostic criteria that consider multiple parameters^3,4^. These criteria include calf circumference, grip strength, SARC_f questionnaire and assessments of limb muscle mass using techniques such as Dual-energy X-ray absorptiometry (DXA) and Bioelectrical Impedance Analysis (BIA). These updated guidelines reflect a more comprehensive approach to Sarcopenia diagnosis, incorporating not only muscle mass but also functional measures and patient-reported performance outcomes.

Numerous studies have investigated the use of cross-sectional area measurements as a proxy for muscle assessment. For instance, the study by Miller et al. revealed that hip joint fracture caused the asymmetry of muscle cross-sectional area and intermuscular adipose tissue in CT scan^5^. In the paper, thigh muscle cross-sectional area (CSA) was less on the side of the fracture by 9.2 cm^2^ (95% CI 5.9, 12.4 cm^2^), whereas the CSA of IMAT was greater by 2.8 cm^2^ (95% CI 1.9, 3.8 cm^2^) on the fractured side. Another study by Jung et al. showed significant reduction in muscle mass in the hip flexor (iliopsoas and rectus femoris) were observed on postoperative CT scans. These findings imply targeted exercise for the hip flexor may be beneficial in rehabilitation of hip fractures^6^. Furthermore, the study by Byun et al. indicated measuring psoas cross-sectional area (PCA) has potential as a diagnostic tool for sarcopenia^7^. Notably, the study found the lowest quintile of PCA was significantly associated with mortality in females, with hazard ratio of 1.76 (95% CI 1.05–2.70, p = 0.017).

By quantifying the muscle area in a single plane, these works aimed to assess muscle size and identify individuals at risk of Sarcopenia. However, this approach has limitations. Sole reliance on cross-sectional area overlooks the complexity of individual muscle groups and fails to capture important aspects such as muscle composition, distribution, and overall performance^8^. Moreover, factors such as participant positioning, limb orientation and the choice of imaging plane can influence the accuracy and comparability of cross-sectional area measurements.

Acquiring the volume of each individual muscle by calculating the annotated segmentation mask is achievable. However, manual segmentation on CT scans is a time-intensive, laborious, and costly task that requires significant effort and expertise. The process often exhibits high variation due to the difficulty of differentiating tissue characteristics. CT scans primarily provides excellent visualization of bony structures and dense tissues, but it has limitations in differentiating soft tissues such as muscles. Muscles have similar radiodensity, making it challenge to distinguish individual muscles based on CT scans. Nevertheless, achieving precise voxel-level segmentation is essential for accurately quantifying each individual muscle's volume and gaining comprehensive insights into muscle performance^9^.

To address these challenges, we propose a deep learning-based automatic individual muscle segmentation approach by using UNETR model. This approach leverages the power of deep learning algorithms to learn intricate muscle features and perform precise segmentation at the voxel level. By automating the segmentation process, our proposed method enables efficient and accurate calculation of each individual muscle's volume.

The objective of our study is to automate segmentation, quantify the volume of individual thigh muscles and examine how each individual thigh muscle contributes to performance and impacts to Sarcopenia. This approach holds great promise for advancing sarcopenia assessment, providing a more comprehensive understanding of muscle quality and performance in individuals affected by this condition.

## Methods

### Study design

In this study, we trained an AI model that segments individual muscles from whole thigh level CT scan and calculates each individual muscle volume for Sarcopenia evaluation. The study utilized a dataset of 72 (train: 60 and validation: 12) whole thigh CT scans obtained from 72 hip fracture patients at Gyeongsang National University Hospital. The dataset was annotated by two radiologists, providing ground truth for training and validation of the AI model. The study adhered to the principles of the Declaration of Helsinki and was approved by the IRB at Gyeongsang National University Hospital. All research procedures were carried out with strict adherence to ethical standards, including protection of participants' privacy, confidentiality, and rights.

In this study, we employ a semantic segmentation model to calculate muscle volume, as discussed in the “Deep learning method of automatic muscle segmentation” section. This model operates by classifying each pixel in an image into distinct categories. In the context of muscle segmentation in CT scans, this means identifying and categorizing each pixel as part of a particular muscle or as background. Capturing intricate details and variations in the images is vital because muscles have complex structures and can vary significantly between individuals. Accurate segmentation requires the model to recognize these subtle differences, ensuring precise analysis and assessment of sarcopenia.

For the segmentation task, we utilized the state-of-the-art deep learning model, UNETR, designed for precise voxel level segmentation and sequential information. This architecture provided the foundation for our AI model to achieve high-quality segmentation results.

The model’s performance was evaluated using various metrics, including dice score (DC), average symmetric surface distance (ASSD), relate absolute volume difference (RAVD), volume correlation (VC) and hausdorff distance (HD)^10,11^. To validate the robustness of the trained AI model, the predicted individual muscle segments were compared with manual segmentations performed by two radiologists. The study design encompassed rigorous statistical analyses to evaluate the performance of the trained AI model and assess its clinical utility in Sarcopenia assessment.

### CT scans acquisition

The study comprised 72 CT scans with annotation from a cohort of 98 participants who had been diagnosed with hip fractures. The mean age of the participants was 77.3 ± 9.73 (standard deviation) years. The CT scans were performed in supine position (Head-First Supine and Feat-First Supine), covering the entire thigh region from hip to knee joint which we call the whole thigh level CT scans. The participants were recruited from Gyeongsang National University Hospital between December 2016 and June 2022.

**Figure 1:**
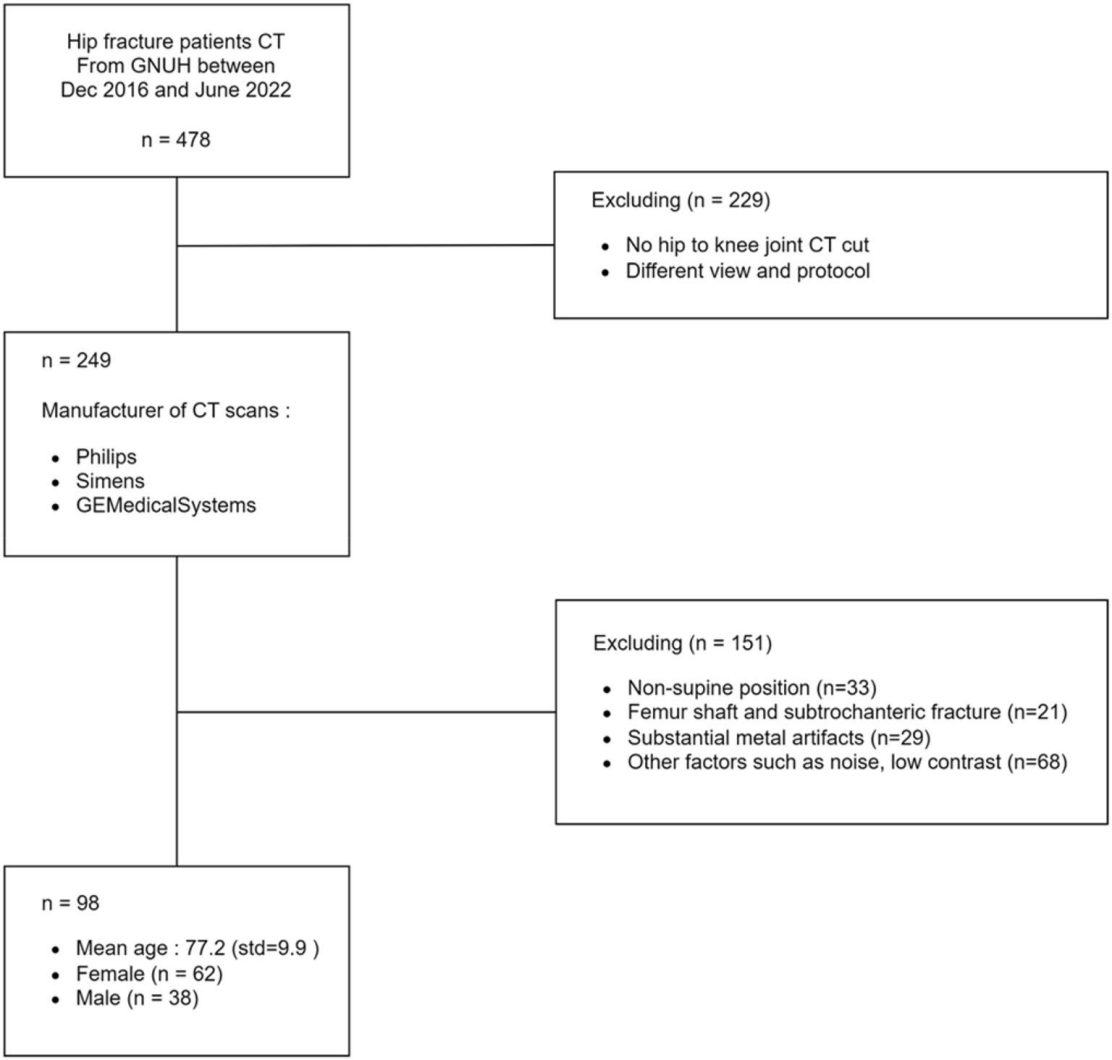
Dataflow. The data flow of the study. In 478 hip fracture patients CT datasets from GNUH between December 2016 and June 2022, the inclusion criteria consisted of age over 50, stable medical condition and no contraindications to CT scanning. Exclusion criteria encompassed such ad missing hip to knee joint cuts, variations in scanning protocols, lower limb amputations, femur shaft fractures, subtrochanteric fractures, muscle or bone deformities, substantial metal artifacts on imaging and other image processing factors that could affect data quality. Out of 478 participants, the final dataset of 98 participants was deemed suitable for the study’s objectives.

As demonstrated in Fig. 1, the inclusion criteria consisted of age over 50 years, stable medical conditions, and no contraindications to CT scanning. To ensure the reliability and accuracy of the muscle segmentation for Sarcopenia assessment, certain exclusion criteria including lower limb amputation, femur shaft fracture, subtrochanteric fracture, muscle or bone deformities and substantial metal artifacts on imaging were applied.

As lower limb amputations cases could introduce significant anatomical variations that would affect accurate muscle segmentation. Participants with shaft fractures were excluded due to the potential muscle distortion caused by the angulation of the two parts of the broken femur observed on CT scans was excluded to minimize confounding factors introduced by the fractured femur. Participants with muscle or bone deformities and substantial metal artifacts on imaging were also excluded, as these factors could interfere with the quality and reliability of subsequent muscle segmentation on CT scans.

By implementing these criteria, we aimed to create a homogeneous study population and minimize potential confounding factors that could affect the analysis of muscle segmentation for Sarcopenia assessment in hip fracture patients.

### CT examinations and ground truth labeling

In this study, we classified 30 classes including iliac, femur and background within 5 major thigh muscle groups on hip to knee joint CT scans (whole thigh). The 5 major thigh muscle groups comprised Anterior, Medial and Posterior thigh muscles, Gluteal region muscle and else.

**Figure 2:**
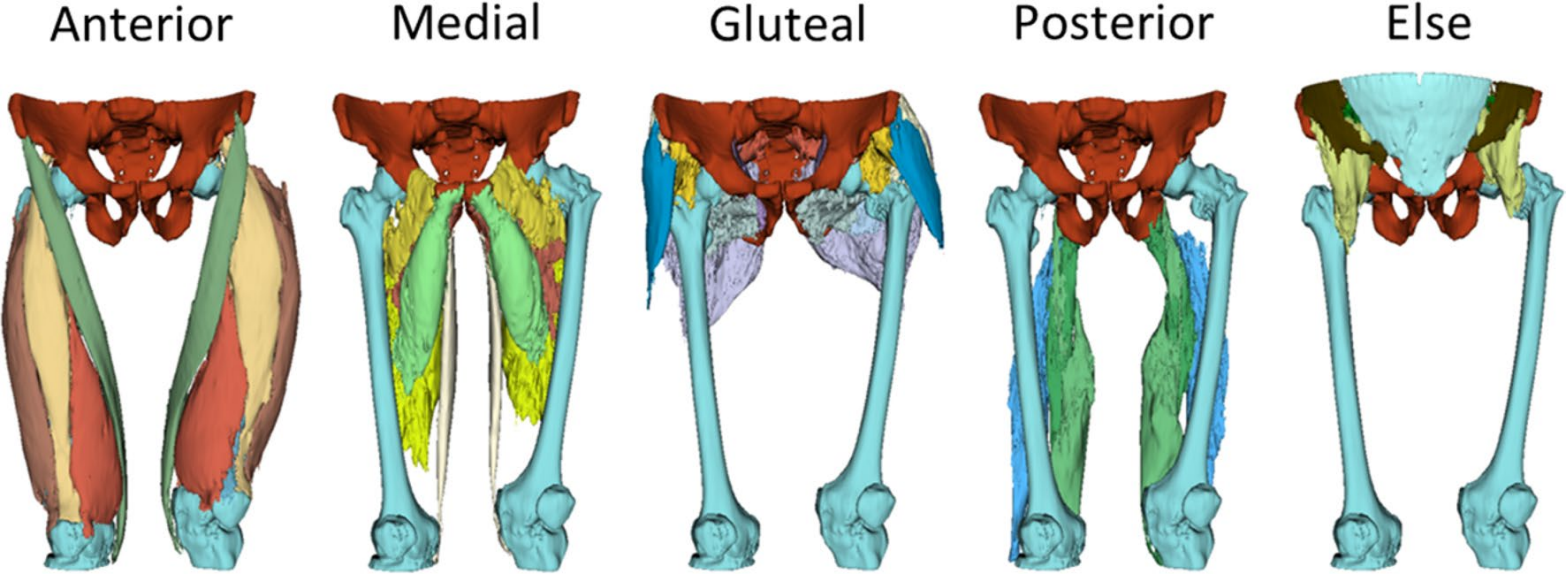
Ground truth image (3D).

The classification of 5 muscles in the Anterior thigh included Sartorius, Rectus femoris and Vastus muscles (lateralis, intermedius and medialis). In the medial thigh, we categorized the 5 muscles as Adductor (magnus, brevis and longus), gracilis and pectineus. The Posterior thigh muscles were classified as Semitendinosus, Semimembranosus and Biceps femoris. In the Gluteal region, the 8 muscles were categorized as Gluteus (maximus, medius and minimus), Fascia latae, Piriformis, Obturator (internus and externus) and Quadratus femoris. Additionally, the Iliacus, Iliopsoas, Psoas, Abdominal oblique, Rectus abdominis, Multifidus, Femur bone, Iilac bone and background on image were classified separately. The example of ground truth image has been displayed in Fig. 2.

The manual annotation process was performed by two experienced radiologists who utilized 3D Slicer and Monai label as annotation tool. 3D slicer, a DICOM viewer program, provides annotation tools such as brush, eraser, and growth from seeds. The growth from seeds method enables the indication of tissue regions based on brushed area in three dimensions and this functionality was developed by 3D slicer. On the other hand, Monai label was employed as server client application, facilitating an efficient annotation workflow by managing the segmentation list and data organization in a regulated manner.

### Pre-processing

**Figure 3:**
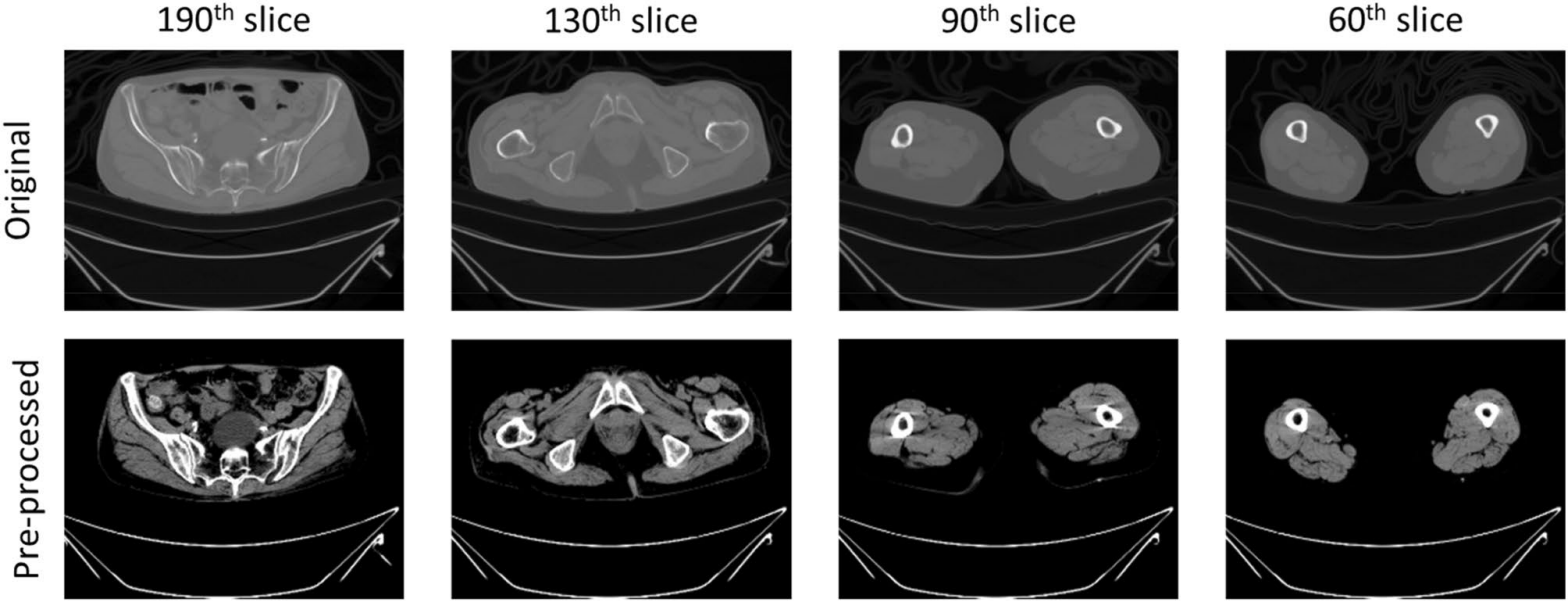
Pre-processing procedure. Presents image pre-processing result.

In the pre-processing phase, several heuristic methods were applied to enhance the deep learning performance in vision tasks. Initially, the intensity range of the CT scan images was scaled from − 57 to 164 to improve the distinction of individual muscle tissues within the CT scans^12,13^. Following this, contrast adjustment was performed using a gamma value of 2, to further enhance the visibility of muscle tissues. The resulting image displayed in Fig. 3 with enhanced contrast was then cropped to only foreground region. The pixel dimensions of the input image and annotation mask were rescaled with scaling factors of (1.5, 1.5, 2.0) using bilinear interpolation for input image and nearest interpolation for annotation mask. Additionally, each image was cropped into four images with a shape of (96, 96, 96) which will be converted into non-overlapping patches of (16, 16, 16) to facilitate the subsequent segmentation process^14,15^. By cropping and rescaling the images, we ensured that the model concentrated on the most relevant areas, thereby reducing the computational burden and enhancing the overall efficiency of the segmentation process.

### Deep learning method of automatic muscle segmentation

**Figure 4:**
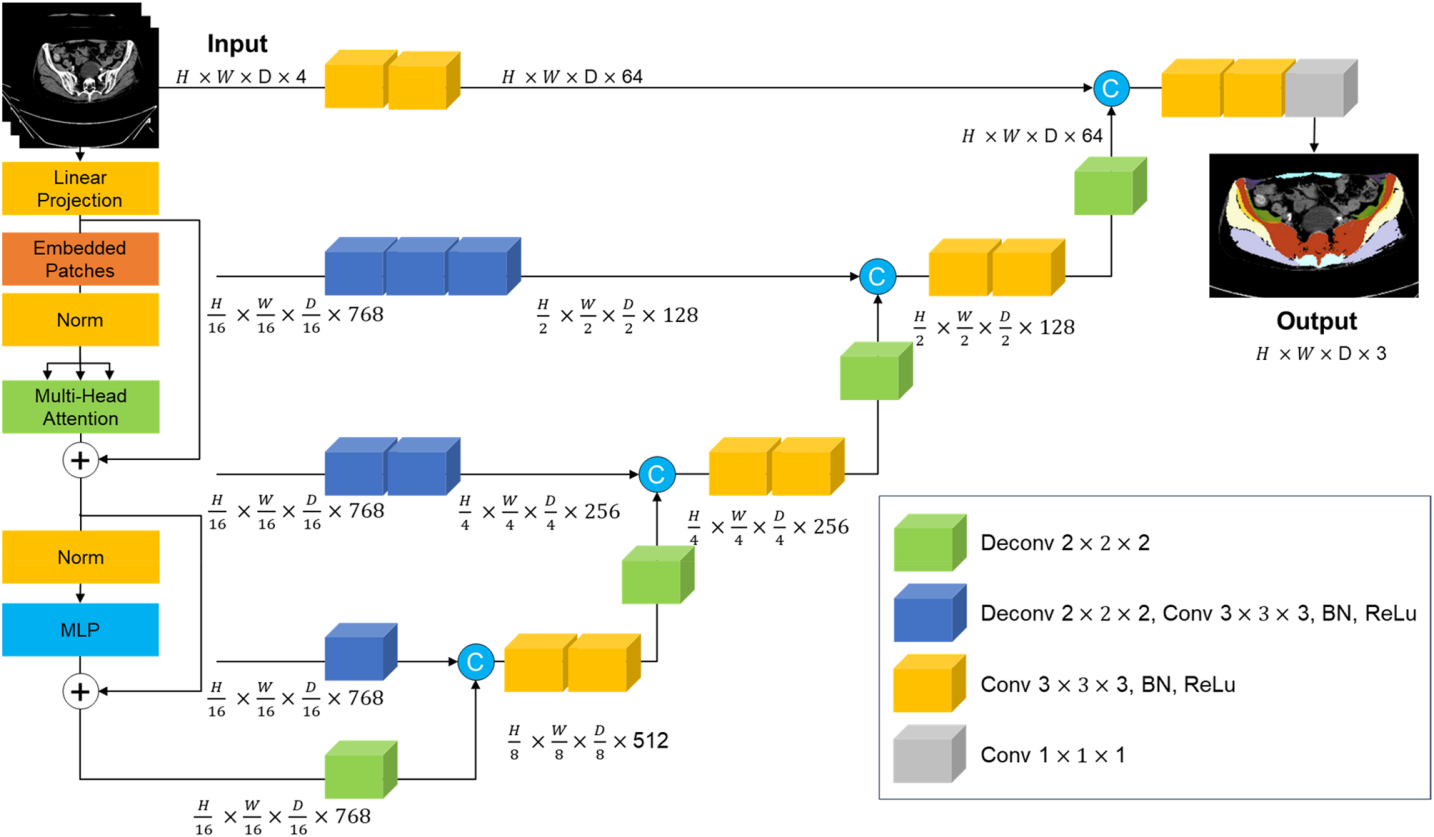
UNETR architecture. Presents overview of the UNETR architecture. This model utilizes the transformer to extract sequence representations from multiple layers and these represents are merged with the decoder through skip connections. The output sizes shown in the figure correspond to a patch dimension of N = 16 and an embedding size of C = 768.

Our study is centered around the task of semantic segmentation which is a crucial task in the field of computer vision, particularly in medical imaging. It serves to delineate and classify different regions of interest within an image^16^. In the context of muscle segmentation, semantic segmentation involves assigning each voxel in the image to a specific class, such as muscle tissue, bone tissue and background. The main challenge in semantic segmentation is the accurate capture of the intricate details and variations in the images, including different patients’ size, position and tissue textures, while also dealing with noise and other imaging artifacts. Due to the ambiguity of each individual muscle tissue in CT scans, our study requires precise voxel-level segmentation^9^. For the automatic segmentation process, we adopted the architecture of the UNETR model demonstrated in Fig. 4^14^.

The UNETR model leverages the power of transformers, which have shown exceptional performance in timeseries domain including Natural Language Process (NLP). The task of 3D medical image segmentation is re-envisioned as a sequence and the UNETR model employs a transformer as the encoder to learn sequence representations of the input volume, effectively capturing global multi-scale information. The encoder follows a U-shaped design, reminiscent of the original U-net architecture, which is known for its success in biomedical image segmentation. The transformer encoder is directly connected to a decoder via skip connections at different resolutions, allowing for the computation of the final semantic segmentation output. This design enables the model to capture both high-level contextual information and low-level spatial details, making it particularly effective for the individual thigh muscle segmentation.

### Training of automatic muscle segmentation model

The training procedure was conducted on a DGX A100 workstation provided by NVIDIA, equipped with Nvidia A100 GPU and running on the Ubuntu 20.04 operating system. The deep learning based segmentation model utilized Pytorch and Monai frameworks for its implementation. During the model training, the dice coefficient was employed as the loss function, which is a common choice for semantic segmentation tasks^17^. The hyper parameters of AdamW optimizer were configured with a learning rate of 8e − 5, weight decay of 1e − 5, batch size of 2 and 5000 epochs. The configuration was determined through a grid search approach, where various values were explored within a learning rate range of 1e − 3 to 1e − 5. The decision of hyperparameters was determined through heuristic techniques, due to their demonstrated capability to achieve the best performance in current datasets for the muscle segmentation model.

### Intraclass correlation coefficient (ICC)

To assess the consistency of the predicted results in medical images, we computed the Intraclass Correlation Coefficient (ICC) value between the manual segmentation masks annotated by two researchers and the predicted masks generated by our proposal model using whole thigh CT scans. The ICC is a statistical measure used to ratings performed on the same subjects or objects. The ICC value based on 95% confident interval, it is generally accepted that the values below 0.5 indicate poor, between 0.5 and 0.75 indicate moderate, 0.75 and 0.9 indicate good and greater than 0.90 are excellent reliability^18^.

By calculating the ICC value, we aimed to determine the level of agreement between the manual annotations and the predictions, thereby assessing the consistency and reliability of the model’s performance.

### Evaluation metrics

In evaluating the performance of our model, we employed several metrics to assess its accuracy, including (1) Dice score (DC), (2) Average symmetric surface distance (ASSD), (3) Volume correlation (VC), (4) Relative absolute volume difference (RAVD) and (5) Hausdorff distance (HD)^10,11^.

The (1) Dice score (DC) measures the overlap between the model's segmentation results and the ground truth, providing a clear indication of the model's accuracy in segmenting muscles. The (2) Average Symmetric Surface Distance (ASSD) calculates the mean distance between the contours of the predicted and actual segmentations, showcasing the model's ability to precisely outline muscle shapes. (3) Volume Correlation (VC) evaluates the correlation between the segmented and actual muscle volumes, demonstrating the model's accuracy in volume estimation. The (4) Relative Absolute Volume Difference (RAVD) sheds light on the volume discrepancies between the model's segmentation and the ground truth, acting as a gauge of the model's consistency in volume representation. The (5) Hausdorff Distance (HD) measures the greatest distance between the surfaces of the predicted and actual segmentations, ensuring the accuracy of the segmentation right up to its furthest points, thus emphasizing the model's capability in accurately defining muscle boundaries.

Each metrics has own advantages and limitations, allowing us to comprehensively evaluate performance of the models in various aspects.

The (1) Dice score, also known as F1 score, quantifies the overlap between the predicted segmentation and the ground truth. It is calculated as twice the intersection of the predicted and ground truth regions divided by the sum of their individual volumes.

$$Dice \;Score= \frac{2TP}{2TP+2FP+FN}$$

The Dice score metric captures both true positive and false positive predictions and is widely utilized due to interpretability. However, Dice score can be sensitive to imbalanced classes, penalizing false negatives more heavily.

The (2) Average symmetric surface distance measures the average distance between the predicted surface and the ground truth surface that it provides insight into localization accuracy.

$$ASSD=\frac{1}{N}\sum_{i=1}^{n}\left(\left|{d}_{i}-{d}_{i}{\prime}\right|\right)$$

$${d}_{i}=Distance \;from \;each \;surface \;point \;on \;the \;predicted$$

$$surface \;to \;the \;nearest \;point \;on \;the \;ground \;truth$$

$${d}_{i}{\prime}=Distance \;from \;each \;surface \;point \;on \;the \;ground \;truth$$

$$surface \;to \;the \;nearest \;point \;on \;the \;predicted \;surface$$

However, the ASSD does not consider volumetric differences and is influenced by outliers.

(3) Volume correlation quantifies the correlation between predicted and ground truth volumes, indicating overall size agreement.

$$Volume \;Correlation=\frac{\left(\mathit{cov}({g}_{p}, {g}_{t})\right)}{\sqrt{\mathit{Var}\left({V}_{P}\right) \times \mathit{var}\left(V{g}_{t}\right)}}$$

The Volume correlation is calculated by using Pearson’s correlation and it’s only calculated with the volume of predicted and ground truth, thus, it tends to ignore the spatial correspondence and may not capture localized errors.

The (4) Relative absolute volume difference measures the percentage of difference between predicted and ground truth volumes, indicating volume estimation accuracy.

$$RAVD=\frac{{V}_{P} -{ V}_{gt}}{{V}_{gt}}\times 100$$

But it also does not capture spatial correspondence.

The (5) Hausdorff Distance measures the maximum distance between predicted and ground truth surfaces, indicating worst-case localization error, so it’s sensitive to outliers, influenced by noise and does not consider volumetric.

$$HD=\mathit{max}\left(h\left(P,G\right), h\left(G,P\right)\right)$$

### Ethical standards

The study adhered to the principles of the Declaration of Helsinki and was approved by the IRB at Gyeongsang National University Hospital. (IRB No. GNUH 2022-01-032-008). All research procedures were carried out with strict adherence to ethical standards, including protection of participants' privacy, confidentiality, and rights.

## Result

### Prediction performance

**Figure 5:**
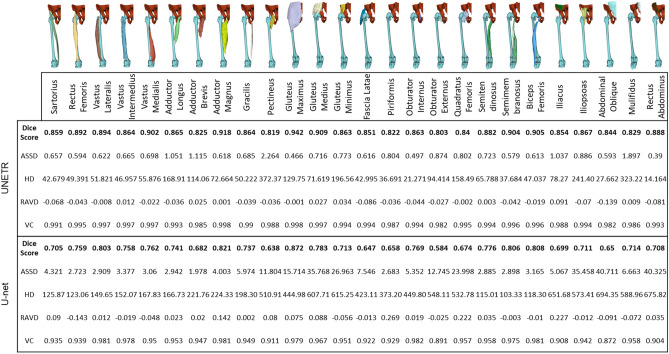
Model performance evaluation. Comparison between proposal model UNETR and baseline model U-net. The table displays average evaluation result of metrics DC (Dice Score), ASSD (Average Symmetric Surface Distance), HD (Hausdorff Distance), RAVD (Relative Absolute Volume Difference) and VC (Volume Correlation) on datasets. This indicates our proposal model UNETR shows higher dice score including other metrics. Notably, the RAVD result suggests that quantifying each individual muscle by using proposal model’s automatic segmentation method is a robust approach.

The prediction performance of our model was evaluated using various metrics through a cross validation method due to limited number of datasets available (60 training samples and 12 validation samples). The evaluation result of the model performance shown in the Fig. 5, among these metrics, the highest average Dice score of each individual thigh muscle class prediction of our model was 0.84 which is 0.1 higher than baseline(3D U-net, 0.69) model for the validation datasets. The Dice score provides a measure of the overlap between the predicted and ground truth. Our model achieved a substantial Dice score, indicating a high level of agreement between the predicted muscle segmentations and the ground truth annotations.

The average ASSD (Average Symmetric Surface Distance) of individual thigh muscle classes was 1.4191 ± 0.91 which is 6.9863 less than baseline (3D U-net, 8.4054 ± 13.36) model for the validation datasets. The Average symmetric surface distance provides insight into localization accuracy between the predicted and ground truth.

Our proposal model achieved an average volume correlation of 0.968 for each individual muscle class in the validation datasets, indicating a high level of accuracy in estimating the volume of each muscle. The Volume correlation metric measures the correlation between the ground truth segmentation mask’s volume and the predicted segmentation mask’s volume. The Volume correlation metric has limitations in capturing local voxel accuracy, as it primarily focuses on the correlation of overall volumes. However, when considering the perspective of each individual muscle volume, the high Volume correlation result demonstrates the robust performance of our model in accurately estimating the volumes of individual muscles. This outcome highlights the model’s ability to provide reliable and precise muscle volume assessments.

**Figure 6:**
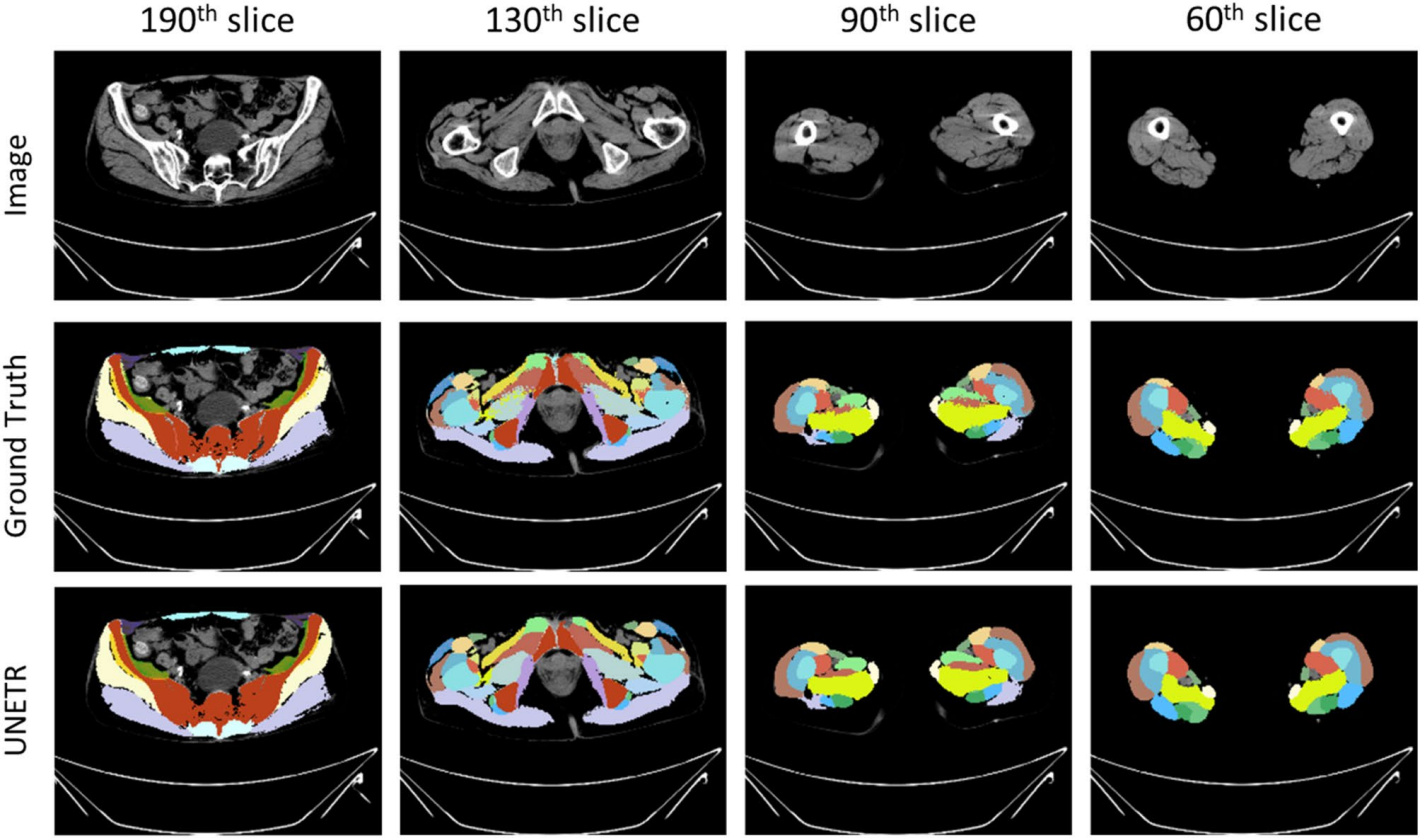
Prediction example image of baseline and proposal model. Example images of segmentation result.

Upon analyzing the result image in Fig. 6, it was observed that the baseline model, lacking sequence information, tended to predict the area of femur area to iliac region. In contrast, our proposal model exhibited accurate predictions in this region. Considering this observation and the difference in Dice scores, our findings suggest that our proposal model outperforms the baseline model in reducing false negative and false positives in local segmentation voxels.

In our hip fracture cohort, we investigated 69 participants and assessed their Skeletal Muscle Index (SMI). The diagnosis of Sarcopenia was made based on the consensus standards, with a cutoff value of 5.4 for females and 7 for males, as defined by the Asian Working Group for Sarcopenia (AWSG2019)^4^. Out of total participants, 41 patients were diagnosed with Sarcopenia (20 females and 21 males), while 28 participants were classified as non-Sarcopenia (23 females and 5 males).

To examine the relationship between Sarcopenia and individual thigh muscle volumes, we calculated the volume of each individual muscle for all participants and adjusted the volume using the SMI calculation formula $(\frac{{mm}^{3}}{{height}^{2}})$ from our automatic segmentation model. We then compared the correlation between Sarcopenia and each individual thigh muscles.

**Figure 7:**
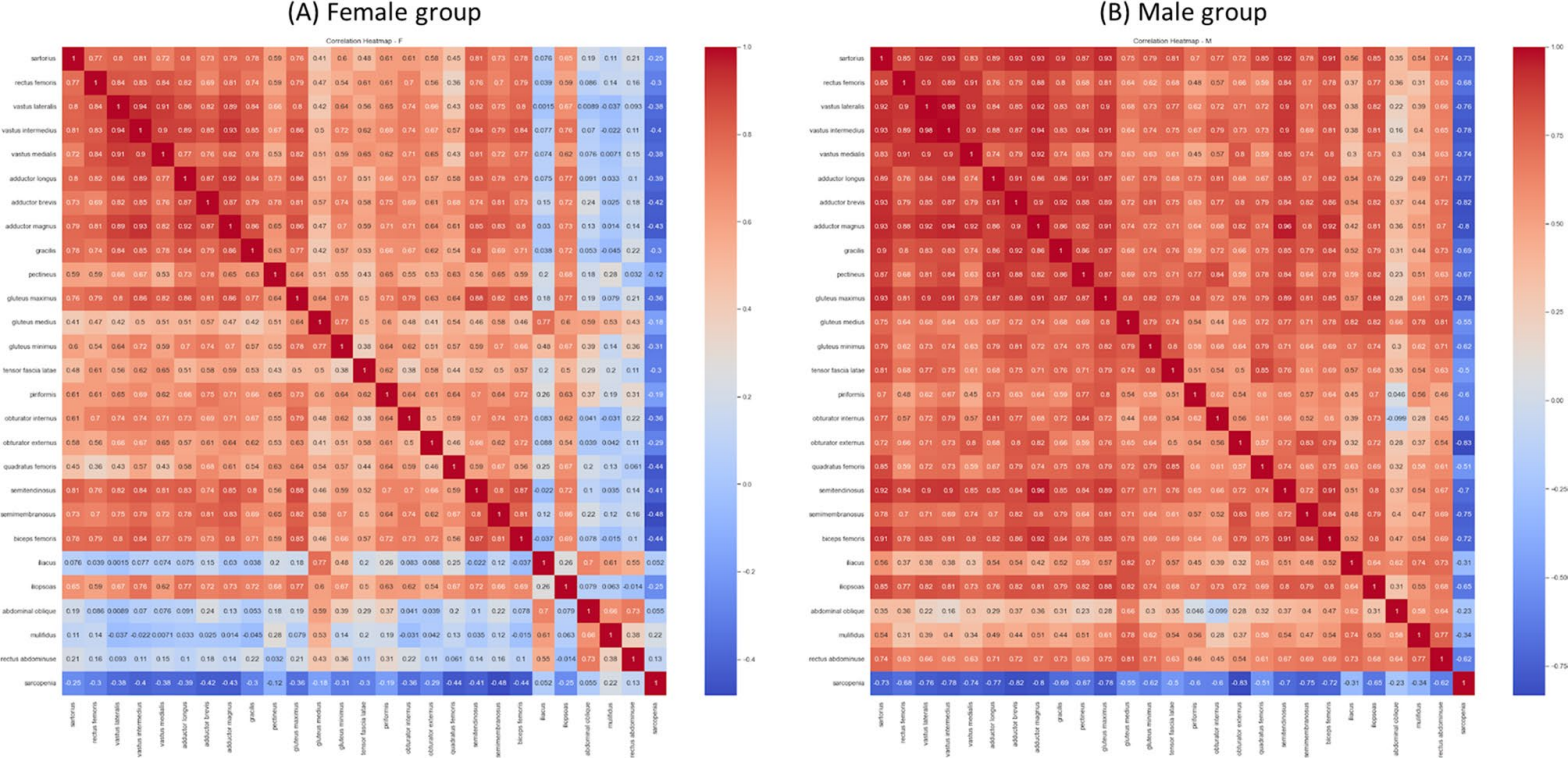
Heatmap between Sarcopenia and individual thigh muscles. Presents a heatmap illustrating the relationship between Sarcopenia and individual thigh muscles grouped by gender. Panel (**A**) represents the female group, while panel (**B**) represents the male group. In the male group, several muscles showed significant negative correlation with Sarcopenia. The sartorius (r = − 0.73), vastus (lateralis r = − 0.76, intermedius r = − 0.78 and medialis r = − 0.74), adductor (longus r = − 0.77, brevis r = − 0.82 and magnus r = − 0.8), gluteus maximus (r = − 0.78), obturator externus (r = − 0.83), semitendinosus (r = − 0.7), semimembranosus (r = − 0.75) and biceps femoris (r = − 0.72) displayed significant correlations.

The result, as depicted in Fig. 7, revealed that in the female group, there were no significant correlations observed between Sarcopenia and the volume of individual thigh muscles. However, in the male group, several muscles showed significant negative correlation with Sarcopenia. These included the sartorius (r = − 0.73), vastus (lateralis r = − 0.76, intermedius r = − 0.78 and medialis r = − 0.74), adductor (longus r = -0.77, brevis r = − 0.82 and magnus r = − 0.8), gluteus maximus (r = − 0.78), obturator externus (r = − 0.83), semitendinosus (r = − 0.7), semimembranosus (r = − 0.75) and biceps femoris (r = − 0.72).

**Figure 8:**
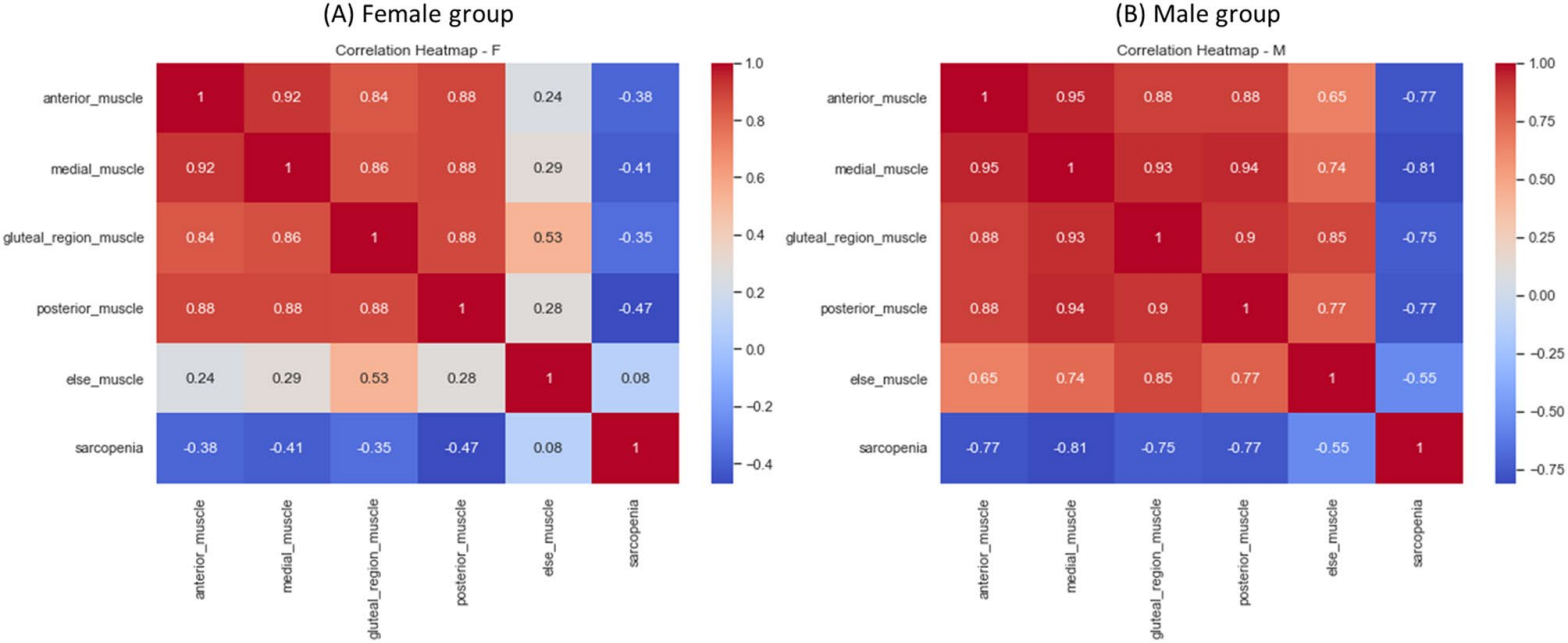
Heatmap between Sarcopenia and thigh muscle groups. Presents a heatmap illustrating the relationship between Sarcopenia and grouped thigh muscles grouped by gender. Panel (**A**) represents the female group, while panel (**B**) represents the male group. In the male group, significant negative correlations were observed between Sarcopenia and the anterior thigh muscle group (r = − 0.77), medial thigh muscle group (r = − 0.81), gluteal region muscle group (r = − 0.75) and posterior thigh muscle group (r = − 0.77).

Furthermore, we investigated the correlation between Sarcopenia and grouped thigh muscles, as illustrated in Fig. 8. In the male group, significant negative correlations were observed between Sarcopenia and the anterior thigh muscle group (r = − 0.77), medial thigh muscle group (r = − 0.81), gluteal region muscle group (r = − 0.75) and posterior thigh muscle group (r = − 0.77).

## Conclusions

### Discussion

As the consensus and research on Sarcopenia shift focus from total muscle volume to muscle performance, our study presents a novel approach to calculating individual muscle volume in the thigh using CT scans. We utilized a deep learning-based segmentation method, which significantly reduced the manual segmentation time of 30 classes on whole thigh CT scan by a factor of almost 900. The average processing time for automatic segmentation per scan was less than 20 s, demonstrating the efficiency of our method and its potential for scaling up to large datasets. The findings presented in the “Result” section suggest that there are notable associations between Sarcopenia and specific individual muscles as well as grouped thigh muscle regions, particularly in the male participants. These results contribute to our understanding of the relationship between Sarcopenia and thigh muscle composition, highlighting the importance of assessing muscle volume in Sarcopenia evaluation.

### Limitation

Our current study, while promising, does present several limitations. The first limitation is inadequate implants and fractures of femur shaft and subtrochanteric. These specific conditions, which are frequently encountered in real-world scenarios, may have influenced the accuracy and applicability of our result. The complexity and variability introduced by these conditions could potentially affect the model’s performance. As a future direction, we plan to enrich our training dataset with a diverse range of cases, including those with implants and other artifacts. This approach will not only enhance the robustness of our model but also extend its applicability to a broader range of real-world scenarios.

The second limitation of our current study is related to label noise. The presence of label noise and diversity in our dataset could potentially lead to less precise segmentation results. This noise is primarily due to time-intensive and laborious manual segmentation process. Furthermore, the diversity in CT scans is attributed to the challenge of distinguishing individual muscle tissues concretely in CT scans. To address this issue, our future work will focus on minimizing the label noise and diversity in our dataset. We intend to employ unsupervised learning methos to detect and rectify inconsistencies in the labels. Through the refinement of our dataset, we aim to enhance the accuracy of our segmentation results and improve the overall efficacy of our model.

### Conclusions

Our study offers a reliable and effective option for segmenting hip to thigh CT scans, enhancing the speed and consistency of calculating the volume of individual thigh muscles. This approach not only streamlines the process but also increases the accuracy of the measurements, making it a valuable tool for researchers and clinicians alike.

Furthermore, we propose the use of individual muscle volume as a quantitative assessment for Sarcopenia evaluation. This approach aligns with the current shift in focus towards muscle performance in Sarcopenia research and has the potential to provide more nuanced and accurate assessments of this condition.

## Data Availability

The data used in this study were collected at Gyeongsang National University Hospital, and inquiries about the data should be directed to the author J.I.Y.
